# Communicating menstrual health in youth sport: insights from Swedish floorball coaches

**DOI:** 10.3389/fspor.2026.1787032

**Published:** 2026-05-14

**Authors:** Flatholm Emil, Tervo Taru, Klingberg-Allvin Marie, Åkerman Eva

**Affiliations:** 1 Department of Women’s and Children’s Health, Karolinska Institutet, Stockholm, Sweden; 2 Sports Medicine, Floorball Research and Development Center, Umeå School of Sports Science, Umeå University, Umeå, Sweden

**Keywords:** adolescent athletes, coaches experiences, coaches perspectives, communicative barriers, menstrual health

## Abstract

**Purpose:**

Floorball, one of the most popular indoor team sports in Sweden, was the focus of this study, which aimed to explore floorball coaches’ experiences of barriers to and opportunities for communicating about menstrual health with female adolescent athletes.

**Methodology:**

An exploratory qualitative study design based on interviews with eighteen floorball coaches coaching girls aged 10–18 years of age was conducted and analysed using thematic analysis. Participants were nine male and nine female coaches aged 31–69 years with experience coaching floorball ranging from 1 to 20 years.

**Findings:**

Coaches experienced inter-linked communicative, educational, and supportive barriers when addressing menstrual health. Many wished to expand their knowledge about menstruation, particularly male coaches who were considered to need greater understanding of the menstrual cycle. Menstruation was generally perceived as a challenging topic for communication due to persistent stigma. Coaches emphasized that while increased communication could help reduce stigma and normalize the subject, discussions about menstruation were still considered sensitive. There was a clear call for organisational support to strengthen education and provide structured guidance for addressing menstrual health. Future research should explore strategies to overcome communicative barriers and how menstrual health education can effectively be implemented in sports.

## 1 Introduction

Menstrual health is defined as a state of complete physical, mental and social well-being and not merely the absence of disease or infirmity in relation to the menstrual cycle (1). To achieve menstrual health, everyone who experiences a menstrual cycle should have access to accurate and age-appropriate information about it, be able to care for their bodies with adequate support, have access to timely and appropriate health care, and live in a positive and respectful environment free from stigma and discrimination (1). Although most people who experience menstrual cycles are women or girls, it is important to acknowledge that not all women and girls menstruate, and that not all people who menstruate identify as girls or women. In this study, when referring to previous research, we consistently retain the original terminology used in the included studies. Menstruation is not only a biological process; it is also shaped by gender norms and social values that influence how it is understood, experienced, and managed. These norms govern what is considered acceptable to say and do, and how bodies are expected to be lived in relation to assigned sex (2). Building on Thorpe et al.'s (2021) feminist poststructuralist framework (3), we approach menstruation as something continuously produced through gendered discourses and norms. In sport contexts, these norms shape how menstruation is understood, talked about, and managed, and they position athletes and coaches in particular ways. Achieving menstrual health also requires eliminating menstruation-related exclusion so that individuals can fully participate in all aspects of life. Despite increasing global awareness of menstrual health as being fundamental to achieving gender equality and human rights (1), menstruation remains a stigmatised topic in many cultures and contexts, including sports (4).

In Sweden, menstrual health is included within the national curriculum through the mandatory area “Sexuality, consent and relationships”, which addresses puberty, reproductive health and bodily autonomy (5). Despite this, recent research shows that stigma, silence and uncertainty around menstruation persist among young people (6). The fact that stigma remains even within a country with comprehensive sexuality education underscores the relevance of examining how menstrual health is addressed within sports settings, where coaches play a significant role in shaping young athletes' experiences. Floorball is one of Sweden's most popular indoor sports with more than 124,000 licensed athletes. Of these players, 37% are female, and among them approximately 58,500 are between 10 and 18 years old (7). Previous research has found that menstrual health literacy is low among female athletes and coaches in terms of knowledge and communication regarding the menstrual cycle and menstruation (8). Considering the substantial number of female participants in floorball in Sweden it is of relevance to examine if this is also evident in this setting. Earlier research conducted among adolescent female athletes showed that the athletes found it difficult to talk about their menstrual cycle due to the dynamics between male coaches and female athletes, a lack of trust, and negative emotions such as embarrassment, awkwardness, fear of judgment, lack of communicative pathways, lack of menstrual cycle specific education and taboo (9). At the same time prior research has shown that female athletes believe that coaches knowledge and willingness to discuss menstruation would strengthen the coach-athlete relationship and reduce menstruation stigma (10). While athletes' experiences of communicating about the menstrual cycle are relatively well understood, there is little research examining the coaches' experiences, especially those coaching female adolescent athletes (11). Prior research has shown that coaches experience a lack of knowledge regarding menstruation (12–16) as well as experiencing communicative barriers on the subject as a result of perceived taboo (14–17). The majority of prior research involving coaches' perspectives on barriers regarding menstruation has been conducted in elite environments (11) further highlighting the need for more research targeting coaches' in non-elite environments, especially in young athletes. No studies have yet examined this within floorball, one of Sweden's largest youth sports, or explored it from the perspective of youth coaches.

In Sweden, 91.4% of menstruating young people aged 16–29 reported menstrual symptoms (18). Physical and emotional symptoms related to the menstrual cycle in young people limit the affected individual's quality of life, social relationships and mental health, as found in a study by Åkerman et al. on menstruating Swedish people aged 18–28 years (6). Abrahamsson et al. found that 19.93% of those aged 16–19 in Sweden reported school absence because of menstrual symptoms (18). Despite the high number of female athletes affected, menstrual health support has been described as limited and inconsistently addressed within this population (17). In a sports setting it has been suggested that educational strategies and new approaches are required for both players and coaches to increase menstrual health literacy in sports (19). To foster a gender-sensitive environment and supportive norms within floorball clubs, there is a need to deepen understanding of coaches' perceptions and experiences of communicating about menstrual health with female adolescent players. Such insights are essential to identify the types of tools and support coaches require to effectively address and support athletes' menstrual health. An increased understanding of both barriers and facilitators to menstrual health communication among coaches is therefore critical. This knowledge can inform strategies to better equip coaches as key actors in promoting menstrual health, reducing stigma, and addressing gender inequities within sport. Therefore, the purpose of this study was to explore the barriers and opportunities for Swedish floorball coaches to communicate about menstrual health with girls aged 10–18 years who play floorball. Additionally, the study aimed to gain a deeper understanding of coaches' knowledge of and attitudes towards issues related to menstrual health. A feminist poststructuralist theoretical lens was applied to analyse how coaches experience communication about menstruation, and how their knowledge and attitudes are shaped.

## 2 Methods

### 2.1 Study design

This is an exploratory qualitative study based on interviews with floorball coaches coaching girls aged between 10 and 18 years. This exploratory, in-depth approach enables an open investigation into the experiences of communication about menstrual health. Qualitative study designs are well suited for examining individuals' perceptions and experiences (20) and offer rich insights into their thoughts and the underlying factors that can guide future interventions to promote menstrual health communication.

### 2.2 Recruitment

The inclusion criteria for participation in the study were as follows: (1) having experience coaching girl teams with ages ranging from 10 to 18 years and (2) being at least 18 years of age. Purposive sampling was used to recruit participants with various backgrounds in terms of sex, coaching experiences (to include non-elite coaches), and coaches with experiences of coaching girls aged 10–18 years. Further, through purposive sampling, we strived to achieve an equal representation of male and female coaches. Recruitment was conducted with support from the Swedish Floorball Federation, which assisted in distributing the information letter to eligible floorball coaches. The information letter contained the contact information of the research team, and the coaches were encouraged to reach out if they had any questions about the study or their participation. Coaches interested in participating in the study were sent written in-depth information about the study as well as a letter of consent. When written consent for participation was given, the interview was planned for.

### 2.3 Data collection

A semi-structured interview guide was used, containing background questions regarding age, experience as a coach, experience coaching girls' teams, the teams’ age ranges, and prior education regarding the menstrual cycle. Furthermore, the interview covered the following topics—availability of and communication about menstrual products, communication with the players about menstruation, perceived barriers to talking about menstruation, needs of menstrual knowledge as coach, suggestions about what clubs can do to develop a “menstrual-friendly” environment and barriers to implementing the suggestions—as well as an open question where coaches could speak freely. Before data collection began the interview guide was tested in one pilot interview and was refined thereafter.

Eighteen coaches were interviewed between June 2024 and September 2024. Data saturation was deemed to be reached when interviews no longer generated new insights or meaningful variation relevant to the study's aim of exploring coaches' experiences of barriers and opportunities for communicating about menstrual health. Saturation was assessed at the level of the overall sample, rather than separately for males and female coaches, as the analysis focused on shared experiences and patterns across the group. Participants were interviewed through Microsoft Teams. The interviews lasted for 10–30 min and were digitally recorded and then transcribed digitally through audio transcription, a software for automatic transcription, and thereafter verified by the author (EF) to assure consistency. If there were inconsistencies between the automatic transcription and the audio file these were corrected by the author (EF).

### 2.4 Participants

Participants from various geographical locations across Sweden were included in the study. Participants were 37–69 years old, nine males with a mean age of 53 (47–69) years and nine females with a mean age of 45 (37–51) years with experiences in floorball coaching ranging from 1 to 20 years (*M* 8.17 years); see Table 1. In addition to the usual teaching in Swedish schools, one participant had taken a course on menstruation in their work setting.

**Table 1:** Descriptives of participants.

Age and coaching experience	Sex	N	Mean (SD)	Min-Max value
Age in years				
	Male	9	52.67 (7.14)	47–69
	Female	9	45.22 (4.15)	37–51
Coaching experience in years				
	Male	9	9.89 (5.13)	4–20
	Female	9	6.44 (3.50)	1–11

SD, Standard Deviation.

### 2.5 Data analysis

A thematic analysis was used to analyse the transcriptions (21). Early analysis was initiated immediately after each interview once the transcript was completed. Insights from the first few interviews prompted minor adjustments to the interview guide to ensure that the questions more clearly aligned with the study's purpose. All transcripts were read several times by the research team to achieve familiarization with the data and to gain a holistic understanding of participants' narratives. During this phase, initial reflections and analytic notes were made and discussed within the research team. The data were coded inductively, with attention to both semantic and latent meanings related to coaches' experiences of communicating about menstrual health. Coding was conducted manually by EF and EÅ, who independently identified meaningful segments of text and assigned preliminary codes. The codes were organized and grouped according to conceptual similarity and relevance to the study’s objectives, using a tabular structure to provide a clear overview during the analytic process. Researchers contributed to the analytic process through active involvement in the development, refinement, and interpretation of themes, ensuring analytical depth and coherence across the dataset. These groupings formed the foundation for developing preliminary themes, which were then visually organised into a thematic map. The thematic map was used iteratively to visualise and refine the relationships between sub-themes and themes throughout the analytic process (21). The preliminary themes were discussed within the research group to define and name the final themes. Throughout the entire process, the coding and emerging themes were ongoingly discussed and refined collaboratively within the research group. There were no major disagreements highlighted during the analytic process within the research group and consensus regarding codes and themes were achieved.

### 2.6 Ethical considerations

Ethical approval was sought from the Swedish Ethical Review Authority (registration number 2024-00691-02). However, according to Swedish law, a committee judgment was not required for the conduct of the study, as it did not include any sensitive data of the study participants. Informed written consent was obtained from all participants before their participation. Participants received both written and oral information about the purpose of the study before the interviews took place. All participants were informed that participation was voluntary and that they could decline to participate or withdraw their consent to participate at any time. They were also informed that their answers would remain confidential and that all identifying variables would be deleted. All participants agreed to have the interviews audio recorded.

## 3 Results

**Figure 1:**
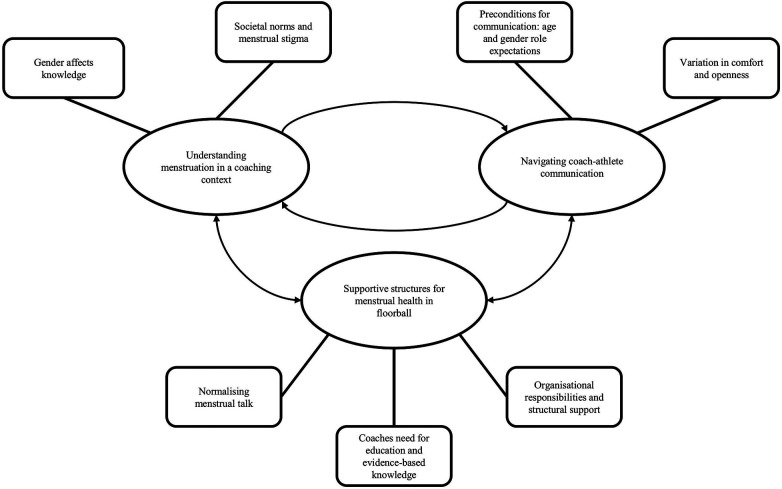
Visual presentation of main themes (circles) and sub-themes (rectangles) as well as how the main themes interlink to one another.

Analysis of the interviews resulted in three main themes: Understanding menstruation in a coaching context, Navigating coach–athlete communication, and Supportive structures for menstrual health in floorball (Figure 1). Each had corresponding subthemes. Figure 1 illustrates that coaches' sex affects their basal knowledge regarding menstruation and that societal norms and menstrual stigma affects how coaches view their ability to communicate with their athletes. We found pre-conditions affecting coach-athlete communication to be age and gender role expectations and that there is an individual variation in comfort and openness when talking about menstruation. Understanding how coach-athlete communication regarding menstruation is taking place resulted in insights into the communicative barriers coaches face and what support coaches need to overcome those barriers. The figure also demonstrates how having supportive structures for menstrual health in floorball plays a key role in facilitating coach–athlete communication about menstruation. Specifically, it shows that coaches identify organisational responsibility for supporting them in increasing their knowledge of menstruation, and that normalising menstrual discussions is perceived as essential for enhancing communication between coaches and athletes.

### 3.1 Understanding menstruation in a coaching context

This theme explores coaches' preconceptions and attitudes regarding menstrual health. Coaches shared the belief that their knowledge regarding menstruation could improve, but in different ways based on the coaches' sex. Male coaches stated they did not have any own experience regarding menstruation while female coaches do. Coaches acknowledged menstruation as a natural aspect of life; however, discussions were influenced by prevailing gender norms and societal stigmas, but menstruation was also thought not to be as sensitive as a subject today.

#### 3.1.1 Gender affects knowledge

Female coaches disclosed having basic knowledge regarding menstruation due to being women, while male coaches acknowledged that female coaches were ahead in terms of knowledge of menstruation since they would never gain any practical experience of menstruation themselves: “*But since I can't have it myself, I don't know how it feels, so I'm just referring to what I've heard”. (male 3)* and “*We have two girls who are leaders on our team and here they are ahead of me. Because they know. That*’*s how it is”. (male 4).* Female coaches also expressed how male coaches lack knowledge regarding menstruation due to not having any own experience: “*Because they don't really know how or men don't really know how it feels and how it can affect training and stuff like that”. (female 18).* Male coaches expressed having a greater lack of basic knowledge regarding menstruation while female coaches disclosed having a greater basic knowledge than males due to being female themselves.

#### 3.1.2 Societal norms and menstrual stigma

Coaches' narratives revealed several examples of how norms and societal stigma impacts how menstruation is addressed. It was a common belief among the coaches that menstruation is a private subject that is not commonly spoken about, even though it's a more open subject today compared to years ago. Coaches agreed that menstruation is a natural part of life that most females' experience and described menstruation as a normal occurrence, but for most of the coaches it remained a stigmatized subject, as expressed by one coach: “*It is the most natural thing on the human body for women. But it*’*s not talked about, it*’*s just there.” (male 3).* Coaches also expressed how menstrual talk probably is further normalized in today's society: “*In today*’*s society I think more people can talk about it.” (female 18)*, and: “*I can imagine that society today with social media and everything is more open today than it might have been 10–15 years ago. Or 20 years ago especially. I think it may be easier for girls today to know how other girls experience things than it was in the 90s or the 20s”. (male 1).* Menstruation was acknowledged as a sensitive subject, to a greater extent for males compared to females, but also that society as of today is more open to menstrual talk and sharing experiences today.

### 3.2 Navigating coach–athlete communication

This theme captures how coaches navigate communication with athletes, including factors influencing the possibility to communicate about menstruation and what coaches expect before initiating communication.

#### 3.2.1 Preconditions for communication: age and gender role expectations

Male coaches expressed that not having any own experience of menstruation, as well as being a man, affected their ability to speak with their players regarding menstruation while female coaches found it easier to initiate communication regarding menstruation with their players. Coaches regarding of gender expressed how barriers communicative barriers are greater towards male coaches than female coaches, as expressed by one coach: “*I think the barrier for the girls is greater to the male leader based on understanding and knowledge. That they think it*’*s embarrassing, that it*’*s hard”. (male 5).* Another coach expressed how communication regarding menstruation takes place differently depending on the presence of a male or female coach: “*The conversation goes a little differently when he*’*s not around. It has nothing to do with him as a person. I'm completely convinced of that. It just has to do with the fact that he*’*s a man, I think”. (female 17).* One coach also described how players would not ask a male coach for menstrual protection but that it was also thought to now be de-dramatized scenario indicating that normalization may be achieved. Coaches expressed beliefs that the age of the athlete may affect how the players choose to communicate about menstruation: “*But they are perhaps at an age where they might not want to talk about it that much yet. But maybe that*’*s because it has not happened yet, the first meeting so to speak”. (male 4)*, as well as the age of the coaches also playing its part: “*It could be, besides the sex, I can imagine that as it is today the head coach is very young. He is only twenty-one, so that is also perhaps a sensitive thing. Maybe it would be easier to bring along another leader who is fifty in that case, who might be more father-like, I would think”. (female 18).* Pre-conditions for communication was thought to be the sex of the coach as well as the age of players and coaches. These factors were expressed by the coaches as potential barriers to communication regarding menstruation.

#### 3.2.2 Variation in comfort and openness

Experiences of one-on-one conversations about menstruation were described by several coaches (7 out of eighteen coaches), most of whom were female coaches. Coaches expressed how different personalities among players affect communication regarding menstruation, some are outspoken and some are more withdrawn: “*It depends entirely on what kind of girls they are too. We have quite a few personalities, so some are more open about it, and some are a bit “hush hush” (female 16)* and “*The girls are quite outspoken and grown up now, so they just come and ask if it*’*s there or shout out loud that someone has it (menstrual protection). It*’*s not strange at all. Then there are probably those who are much more withdrawn and who wouldn't have been able to ask”. (female 18).* Coaches also expressed a perceived individual variance regarding how much information players want to share: “*There must also be respect for the fact that all people are different and how much they want to share”. (Male 6).* Coaches believed that variation in openness regarding menstrual talk may be related to the player’s pubertal phase, as expressed by one coach: “*They are probably also in a transitional period in their own lives. They are certainly somewhat wise, but they are likely in different phases on an individual level, which means that they might not even as individuals want to talk with others in the group about it and such for some reason, I can imagine that. Someone was maybe early and someone is late and I do not know”. (male 8).* Menstrual talk was thought to be a sensitive due to varied reasons during different pubertal phases, that some may think that it is embarrassing to have their menstrual debut and that it may also be embarrassing not to have gotten it too.

Apart from individual variations among players coaches also expressed individual variations in openness among the coaches themselves. One male coach described how he would not feel comfortable talking about menstruation with their players “*But like I said, it*’*s not easy. I think it*’*s a bit like that. I don't know if I would feel comfortable doing it, I should do it”. (male 10)*. Another coach expressed how everyone does not always feel comfortable speaking about menstruation but that it can change over time: “*I think that if someone doesn't want to talk about it, we shouldn't simply force it. You have to start from how comfortable you are and grow into that role too”. (female 7).* Coaches described how openness regarding menstruation varies individually among the players as well as the coaches due to a variety of personalities. It was also believed that the players' pubertal phases may affect how communication regarding menstruation takes place.

### 3.3 Supportive structures for menstrual health in floorball

This theme captures the needs the coaches expressed in terms of improving their work with menstrual health. Coaches desired to normalize menstrual talk and asked for more education and support from their respective organizations locally and nationally.

#### 3.3.1 Normalising menstrual conversations

Coaches agreed that menstruation should not be a sensitive subject in a sports setting. It was described how there is a need to normalize menstrual conversations among players as it's a normal occurrence in their lives: “*You need to lower the threshold for the girls, what should I say..* *It is a part of life for the girls and somewhere it should not be an obstacle, like in training or matches”. (male 5).* One coach expressed that communication regarding menstrual health should be just as normalized as a player telling their coach they are sick: “*As well as being sick for other reasons, you have to be able to say that today I was like one. I have a lot of period pains”. (male 6),* and that coaches regarding of their sex should be able to communicate about menstruation to de-stigmatize the subject: “*I think everyone should talk about it. Everyone should. It shouldn't be something; some taboo or that it*’*s just girl related. I also think it*’*s important that guys and male coaches like female coaches need to kind of reduce this taboo or that it would be disgusting or something scary or weird or something”. (female 17)*. One coach also expressed that that the more you address menstruation the more normalized it becomes: “*But I think that if you talk about it, it*’*s like everything, if you talk about it, it usually becomes normal”. (male 3).* Coaches expressed how menstruation should be a normalized topic within a sports setting and that players should not experience barriers to initiate communication regarding menstruation, such as requesting menstrual protection. To achieve this, coaches expressed belief that stigma and taboo need to be reduced mainly due to normalizing communication towards the subject.

#### 3.3.2 Coaches' need for education and evidence-based knowledge

Coaches expressed desire and need for more education regarding menstruation. Males expressed a greater need for basic education while female coaches, due to their own experience of menstruation, were thought to have a greater basic knowledge. But regardless of sex it was thought that as a floorball coach for girls you should have basic knowledge about menstruation: “*Basics maybe, I think everyone should know. I also think that if you are a girl or a woman yourself, you automatically already know a lot about it. But as a floorball coach, regardless of sex, I think it is good to know what menstruation is, how it works, approximately when you usually get it, how often you have it, how long you have it and that you need protection. Basics as well”. (female 17).* Several male coaches expressed how their basic knowledge regarding menstruation is low and that they are willing to learn more about the subject. Female coaches also expressed desire to gain more knowledge but asked more specifically about how menstruation affects performance. However, there were both male and female coaches expressing no need nor desire for further knowledge as well as the need and desire for basic and performance specific knowledge regarding menstruation. Coaches also expressed how they wanted the knowledge to be evidence-based since there are many different informative sources: “*I think it*’*s interesting to hear about what the latest advice is on managing menstrual symptoms linked to exercise. I think there*’*s a lot of research being done on that and it*’*s interesting to know. There*’*s a lot of rumors”. (female 13).* The coaches in general desired more education in terms of basic menstrual knowledge as well as how menstruation is linked to performance and exercise. Male coaches also expressed the thought of increased menstrual knowledge as a positive thing, but it was not always clear what the meaning of it is.

#### 3.3.3 Organisational responsibilities and structural support

Coaches disclosed the need for a greater organizational support regarding ways to improve their menstrual work and expressed how a lot can be done if an organization highlights a topic, as already done regarding knee control which is widely incorporated within floorball: “*The floorball association has been very good at highlighting knee control, and I think that quite a lot can be done if you want. You can get an entire organization to see a problem and prevent a problem too”. (male 8).* Coaches also expressed that the various floorball associations need to be the one to take action to incorporate and improve menstrual health work among coaches and that menstrual health should be a part of or offered as a part of education provided by the associations, especially for coaches working with girls: “*If you are going to work with girls, you should be able to go and choose that education”. (female 18)*. Coaches were of the beliefs that menstrual health needs to be highlighted within the floorball association and that it should be just a normal of a subject as injury prevention, especially when coaching girls.

## 4 Discussion

The purpose of this study was to explore barriers and opportunities for Swedish floorball coaches to communicate about menstrual health with young girls aged between 10 and 18 years who play floorball. Moreover, the purpose was to gain an increased understanding of coaches' knowledge and attitudes towards issues related to menstrual health.

Key findings revealed that youth floorball coaches in general expressed a desire to increase their menstrual health knowledge and that coaches would benefit from targeted education. Education should cover basic menstrual knowledge as well as how menstruation affects performance. Menstruation was largely perceived as a natural part of being a female; however, experiences and attitudes towards menstruation vary among coaches. The topic was commonly described as difficult to address, shaped by prevailing stigma and requiring sufficient knowledge and confidence to manage appropriately, to a greater extent for male coaches than for female coaches. Communication about menstrual health was viewed as a potentially powerful way to reduce stigma and normalise the topic, although some coaches acknowledged that it could remain sensitive in certain contexts. It was also believed that today's society is more open for discussions about menstrual compared to years ago. It can be hypothesized that this thought shift in openness may help if menstrual education is to be implemented within the sports setting. Importantly, participants expressed a clear wish for greater organisational support at both the national and regional levels to strengthen menstrual health education and provide structured guidance for coaches.

### 4.1 Expanding coaches' menstrual health knowledge: A priority for development

Coaches described a desire to expand their menstrual knowledge to better meet the needs of their young athletes in a supportive and informed manner, which is consistent with previous research. In a similar context to our study Donnelly et al. examined Scottish youth coaches ability to support adolescent soccer players through their menstrual cycle and found that there were communicative barriers, that there was a lack of menstrual knowledge but yet willingness to learn more about menstruation (16). Low knowledge about menstruation among coaches may lead to an inability or low willingness to address menstruation-related issues with athletes (15). At the same time, it has been found that athletes desire support regarding their menstrual cycle (4). This becomes somewhat paradoxical since coaches are encouraged to address menstruation with their athletes but may not have the required knowledge to address the matter adequately (4, 16). As one coach in our study described it – “It’s just there” – even though most female athletes experience symptoms of menstruation that affect their performance. As already shown (19), it is important to find strategies to improve menstrual knowledge among coaches to enable them to support their athletes. Therefore, further research is recommended on how to implement strategies to address menstrual health in youth athletes and to evaluate the effects of such strategies in practice. Addressing this knowledge gap is critical, as it represents a first step towards breaking menstrual stigma, normalising conversations about the menstrual cycle in sports, and fostering equitable participation opportunities for girls. Our findings demonstrate that menstrual knowledge among coaches is a subject for development.

### 4.2 Normalising menstruation through coach–athlete communication – a powerful way to reduce stigma

Our study underscores that coaches encounter multiple communicative barriers when addressing menstruation with their athletes. These barriers stem from limited menstrual health knowledge, perceptions of gendered expectations and feelings of discomfort or inadequacy when raising the topic. Male coaches described heightened hesitancy to initiate conversations because of concerns about making athletes uncomfortable.

This finding aligns with previous research demonstrating that coaches of adolescent soccer players experience similar communicative barriers, such as limited menstrual knowledge and not knowing how to communicate regarding menstruation (16). The coaches in our study also believed that the communicative barriers were greater for male coaches than female coaches. Similar findings were found among youth soccer coaches, who believed that female coaches are more likely to be trusted with information regarding players' menstruation than male coaches. This is because the athletes are more comfortable with female coaches, the female coaches have personal experience, and the conversation is less awkward in a female–female setting (16). It has previously been found that female athletes find it less comfortable speaking about their menstruation with male coaches (22) due to a lack of trust, embarrassment, awkwardness, fear of judgment, a lack of communicative pathway, insufficient education and taboo (9). Studies have also shown that athletes find it difficult in general to speak about menstruation due to awkwardness (4), shame and taboo (23). Donelly et al. presents that 45% of adolescent football players (aged 15 + −1 year, *n* = 65) found it challenging to communicate about their menstrual cycle (4). This is already a low number, especially considering that almost 90% of the participants experienced menstrual symptoms and 78% of them perceived that their menstruation impacts performance (4). It quickly becomes counterproductive if menstruation is known to affect adolescent athletes but remains an embarrassing, awkward and taboo subject, preventing it from being discussed openly. Our study further highlights that gender stigma regarding menstruation occurs in the male coach–female athlete relationship in a floorball setting. Therefore, menstrual communication barriers and the male coach–female athlete relationship should be further researched in addressing menstrual health.

Our study also highlights coaches' beliefs that the athletes' current pubertal phase may impact how menstruation is communicated. It was expressed that the athletes are in the age of transition and that athletes' pubertal phases vary individually. This may result in communicative barriers for varied reasons during different pubertal phases, as expressed by coaches, that some may think that it is embarrassing to have their menstrual debut and that it may also be embarrassing not to have gotten it too. It should be further examined how athletes in different pubertal phases perceive communication regrading menstruation within a sports setting. These findings highlight the need for policies that support age appropriate communication about menstruation within sports contexts, aligning by recommendations by Hennegan et al. that emphasize the importance of providing age-appropriate menstrual health information (1).

### 4.3 Organisational responsibility: creating conditions for menstrual health communication

Our study presents that Swedish youth floorball coaches express a desire to learn more about menstrual health and how to address the subject adequately. A similar desire among coaches to be able to support their athletes has previously been presented in different contexts (16). A barrier to increasing coaches' knowledge was that they felt they did not have enough support to do so. Prior research has shown that coaches believe that topics considering female biology are not discussed enough and that educational programmes should be expanded (24). Coaches asked for the implementation of solutions by their respective boards or by the floorball federation regarding how to work with menstrual health. Prior research has shown that there is often a lack of clear guidelines regarding how to address menstruation within sports organisations (25) but it’s also been shown that relatively small interventions can increase menstrual knowledge and confidence in coaches (26). Therefore, further research should examine how sports organisations in Sweden may offer support and education to coaches working specifically with female athletes.

## 5 Strengths and limitations

One of the strengths of this study is the involvement of a multidisciplinary research team in analysis, comprising four researchers with varied professional backgrounds and perspectives (sports medicine, physiotherapy, midwifery, reproductive health, and public health). This investigator triangulation allowed the data to be viewed from multiple angles and enriched the data analysis and interpretation, which strengthened the study's credibility (27). Further, using quotes from participants also strengthened the credibility and transparency of the study. Dependability was strengthened by our effort to provide a clear and transparent description of the study process, encompassing data collection, analysis and the presentation of results (27).

Another strength of the study is the inclusion of both female and male coaches, which enabled a more nuanced understanding of gendered experiences related to the study topic. However, there are several limitations concerning the transferability of the findings—that is, the extent to which the results can be applied to other groups or contexts (27). Specifically, we did not ask participants whether they identified as transgender or non-binary or about any self-reported experiences of disability—factors that may shape perspectives and experiences related to the research topic (1). The lack of representation from these groups limits the transferability of the study findings and our ability to capture diverse perspectives within sports settings. We recommend that future studies specifically include coaches with trans and non-binary identities and those with experiences of disability, as they may offer valuable insights into how to foster a more inclusive menstrual health environment in sports settings. Furthermore, the present study included only coaches with experience in floorball, which may limit the transferability of the findings to other sports. Menstrual health is an increasingly recognized topic which may impact the social desirability regarding our study. Informants were recruited via self-selection which may have attracted informants with pre-interest or pre-understanding regarding the subject, this may have skewed the results. A question regarding pre-understanding was asked and only one of the eighteen informants had undergone further education regarding menstruation apart from the obligatory school-education. Regarding cultural context our study was conducted in Sweden where basic education about menstruation is generally expected to be provided in schools. It is unclear to what extent and how consistently this education is provided, as the terms “menstruation’ and “menstrual cycle’ are not mentioned in the school curriculum. However, several findings related to menstrual communication and knowledge were similar to those reported by coaches in other countries (4, 9). Moreover, experiences of menstrual stigma and taboo did not appear to be confined to a specific context or country, although their manifestations may vary across settings. Another limitation is that our study solely focuses on the coaches' perspectives. It is not known if the athletes in this given context perceive communication about menstruation in similar ways as their coaches.

## 6 Implications for policy, practice, and research

Upcoming research should focus on strategies to overcome the barriers coaches experience when communicating about the menstrual cycle, as well as on addressing other aspects of menstrual health. One such strategy is to enhance coaches' menstrual health knowledge; therefore, further research is needed on implementing menstrual health education within the floorball context and on identifying factors that may facilitate this process. Research should also focus on how to involve male coaches to a greater degree in the promotion of menstrual health in sports settings and on addressing the economic perspective regarding the implementation of strategies.

### 6.1 Practical implications

- Integrate menstrual health into coaching education: National and regional sports federations should embed menstrual health modules into coaching certification programs and continuing education, with a focus on symptom recognition, communication strategies, and inclusive practices.
- Provide accessible, evidence-based resources: Develop short, practical guides and online resources for coaches that explain menstrual cycle basics, performance impacts, and management strategies, making knowledge easy to access and apply.
- Normalise coach–athlete communication: Encourage coaches to initiate non-stigmatising, open conversations about menstruation with their teams. This can help normalise the topic, reduce athlete anxiety and foster trust.
- Strengthen organisational support: Clubs and federations should create policies and allocate time during training to address menstrual health, ensure menstrual products are available at facilities, and signal institutional commitment to inclusion.

## 7 Conclusion

This study highlights that floorball coaches recognise the importance of menstrual health, but face barriers related to limited knowledge, gendered expectations, and discomfort in initiating conversations with athletes. Coaches expressed a strong desire for increased education, particularly male coaches, and emphasised that open communication could reduce stigma and improve athlete well-being.

The findings underscore the need for organisational support and structured educational initiatives at the club and federation levels to equip coaches with the knowledge and confidence to address menstruation effectively. Addressing these gaps is a critical step towards normalising menstrual health discussions, reducing stigma, and promoting equitable participation opportunities for adolescent girls in sports.

Our findings increase the understanding of floorball coaches' experiences of inter-linked communicative, educational, and supportive barriers to promoting menstrual health. Once again referring to menstrual health, a definition for policy, practice, and research to achieve menstrual health should be prioritised moving forward. Understanding how to implement strategies to overcome barriers experienced by coaches in communicating about menstrual cycles is important to further improve menstrual health in young athletes.

## Data Availability

The raw data supporting the conclusions of this article will be made available by the authors, without undue reservation.
